# Direct Smear Versus Liquid-Based Cytology in the Diagnosis of Bladder Lesions

**DOI:** 10.30699/IJP.2021.528171.2646

**Published:** 2021-11-15

**Authors:** Mahmoud Reza Kalantari, Mohammad Ali Jahanshahi, Masoumeh Gharib, Sara Hashemi, Shakiba Kalantari

**Affiliations:** 1 Department of Pathology, Mashhad Faculty of Medicine, Mashhad University of Medical Sciences, Mashhad, Iran; 2 Students Research Committee, Mashhad University of Medical Sciences, Mashhad, Iran

**Keywords:** Bladder Cancer, Direct Smear, Liquid-Based Cytology, Urine Cytology

## Abstract

**Background & Objective:**

Urine cytology is an important diagnostic method for urinary tract cancers (especially carcinomas), which is suitable for follow-up of residual urothelial tumors after surgery of malignant bladder tumors. Liquid-based cytology (LBC) was used for the first time in cervical cytology Compared to direct smear cytology (DSC), LBC reduced background elements (including cellular debris, inflammatory cells, and blood cells), provided better cell preservation, and had a higher satisfaction rate. In this study, we performed two different methods (DSC and LBC) to detect bladder lesions; also, we determined the sensitivity and specificity of these methods.

**Methods:**

A total of 146 samples were taken from patients with suspected bladder cancer and processed for direct smear and LBC. In both methods, findings were reported according to the Paris System. Then, patients underwent cystoscopy and biopsy. Next, the accuracy of cytology methods was evaluated according to biopsy reports. The sensitivity and specificity of these methods were also calculated.

**Results:**

Credit indices obtained for the direct smear method included sensitivity (62.5%), specificity (89%), positive predictive value (89.5%), and negative predictive value (91.5%). For LBC methods, credit indices included sensitivity (85.7%), specificity (99%), positive predictive value (96%), and negative predictive value (96%). Agreement between the two methods was statistically significant (*P*<0.000) in negative biopsies but not in positive biopsies (*P*>0.05).

**Conclusion:**

This study showed that LBC has higher sensitivity and specificity than the direct smear.

## Introduction

Bladder cancer is one of the most common cancers of the urinary system (1). Bladder urothelial cell carcinoma is a heterogeneous group of tumors with different malignant potentials (2, 3). Approximately 80% of bladder cancers are low-grade superficial tumors (4). Although superficial tumors are resected transurethrally, the recurrence rates of these tumors are high (60%-85%) (5). Fortunately, the five-year survival rate is high (80%-90%) (6), and that is why the biggest concern in patients with superficial bladder cancer is to reduce and procrastinate the recurrence and prevent the progression to invasive disease (7). Therefore, long-term follow-up is needed in these patients. Routinely, cystoscopy and cytology are used to diagnose and follow up superficial bladder tumors (8). Cystoscopy is the most efficient method available to detect primary or recurrent bladder cancer (9). Yet cystoscopy is an invasive procedure and causes some discomfort in patients, and it might be ineffective in diagnosing in situ or superficial tumors (9-11). Therefore, it is important to use urine cytology as a noninvasive complementary method (12, 13). Urine cytology is an important noninvasive diagnostic method for urinary tract cancers, especially carcinomas (13, 14). It has a 95% sensitivity and nearly 100% specificity in detecting high-grade urothelial malignancies (15). However, it is a low-sensitive method for detecting low-grade malignant urothelial tumors (the most common urothelial carcinoma) (16, 17). It is useful for follow-up in treated patients and to evaluate the residual of malignant bladder tumors after surgery (18). One of the recent methods is liquid-based cytology (LBC), which was used in cervical cytology for the first time (19). Urinary samples for LBC were centrifuged, and then the precipitates were transferred to a Cytolytic solution (Sina Dej Sahand Co, Tabriz, Iran). After the second round of centrifugation, two or three drops of precipitates were transferred to a preservative solution (Sina Dej Sahand Co, Tabriz, Iran). The vial and slide were placed in a ThinPrep processor (SHANDON, CYTOSPIN3, UK). Then, the preparation steps (i.e., dispersion, cell collection, cell transfer, and staining) were done.

Compared to direct smear cytology (DSC), LBC has lower background elements (such as cellular debris, inflammatory cells, and blood cells), provides better cell preservation, and has a higher satisfaction rate (20-22). In the LBC method, after adding the fixative solution, all extracted cells were maintained; therefore, there are more cells for cytological examination.

The purpose of this study was to evaluate urine cytology, detect bladder lesions with two different methods (i.e., direct smears and LBC), and determine the sensitivity and specificity of these methods.

## Material and Methods

A total of 146 patients suspected of having bladder carcinoma were selected from Armaghan Urology Clinic, and their urine specimens were examined by two cytology methods (direct smear and LBC); also, their sensitivity and specificity were compared. The appropriate sample size was estimated from previous studies (20, 23). 

All urine samples were collected at midday to prevent false-positive results (morning samples were not taken). The patients were advised to drink a few glasses of water 1-2 h before sampling. All samples were freshly voided urine. Urine samples were taken in our laboratory or immediately transferred to our laboratory; instrumented urine specimens were excluded. The gross nature of samples (such as color, volume, and clarity) was recorded. In our laboratory, each specimen was divided into two halves for further processing. One-half of the voided sample was prepared for the direct smear method and another half for the LBC method. To prevent cell damage, 10% formalin or 50% alcohol was equally added to the sample size immediately. 

In the direct smear method, slide preparation was done by sediment obtained from centrifugation of urinary samples (1000 rpm for 30 min). The slide was stained with Papanicolaou and hematoxylin and eosin (H&E) staining methods.

In LBC, samples were centrifuged at 1500 rpm, and the precipitates were transferred to 30 mL of a Cytolytic solution (Sina Dej Sahand Co, Tabriz, Iran). After another round of centrifugation, two or three drops of precipitates were transferred to the preservative solution (Sina Dej Sahand Co, Tabriz, Iran). The vial and slide were placed in a ThinPrep processor (SHANDON, CYTOSPIN3, UK). The preparation steps are as follows: 1) dispersion: filter rotates within the sample vial, creating currents in the fluid that are strong enough to separate debris and disperse mucus but gentle enough to have no adverse effect on cell appearance; 2) cell collection: a gentle vacuum is created within the ThinPrep Filter, which collects cells on the exterior surface of the membrane; 3) cell transfer: after collecting the cells on the membrane, the ThinPrep Filter is inverted and gently pressed against the ThinPrep Microscope Slide. Natural attraction and slight positive air pressure cause the cells to adhere to the ThinPrep Microscope Slide, resulting in an even distribution of cells in a defined circular area. Then, ethanol 95% is used for fixation; and 4) staining: Papanicolaou and H&E staining methods were performed manually.

In both methods, the findings were reported according to the Paris System. Thereafter, all patients underwent cystoscopy and biopsy, and the precision of both cytology methods was compared according to biopsy results. 

This study included all patients with suspicious symptoms and signs of bladder carcinoma or recurrent disease and all those patients with a history of bladder carcinoma referred for follow-up studies. We excluded patients with incomplete cytology, cystoscopy, and biopsy procedures from the study. Data collected from clinical and laboratory observations were analyzed using SPSS 21 (SPSS Inc., Chicago, Ill., USA). The clinical and laboratory characteristics of patients were described by descriptive statistical methods, and Student *t*-test, chi-square, McNemar, and Kappa tests were used. The sensitivity, specificity, positive, and negative predictive values of direct cytology and liquid-based methods were also calculated and compared based on final biopsy results.

All patients went through routine diagnostic and therapeutic procedures, and no additional diagnostic procedures were performed.

Here are some cytologic criteria that were used in our study to report:

Low-grade urothelial neoplasia (LGUN): three-dimensional cellular papillary clusters (defined as clusters of cells with nuclear overlapping, forming papillae) with fibrovascular cores, including capillaries. 

Suspicious for high-grade urothelial carcinoma (SHGUC): this diagnosis is restrictively used in cases with abnormal urothelial cells that quantitatively fall short of a definitive diagnosis of high-grade urothelial carcinoma (HGUC).

**Fig. 1:**
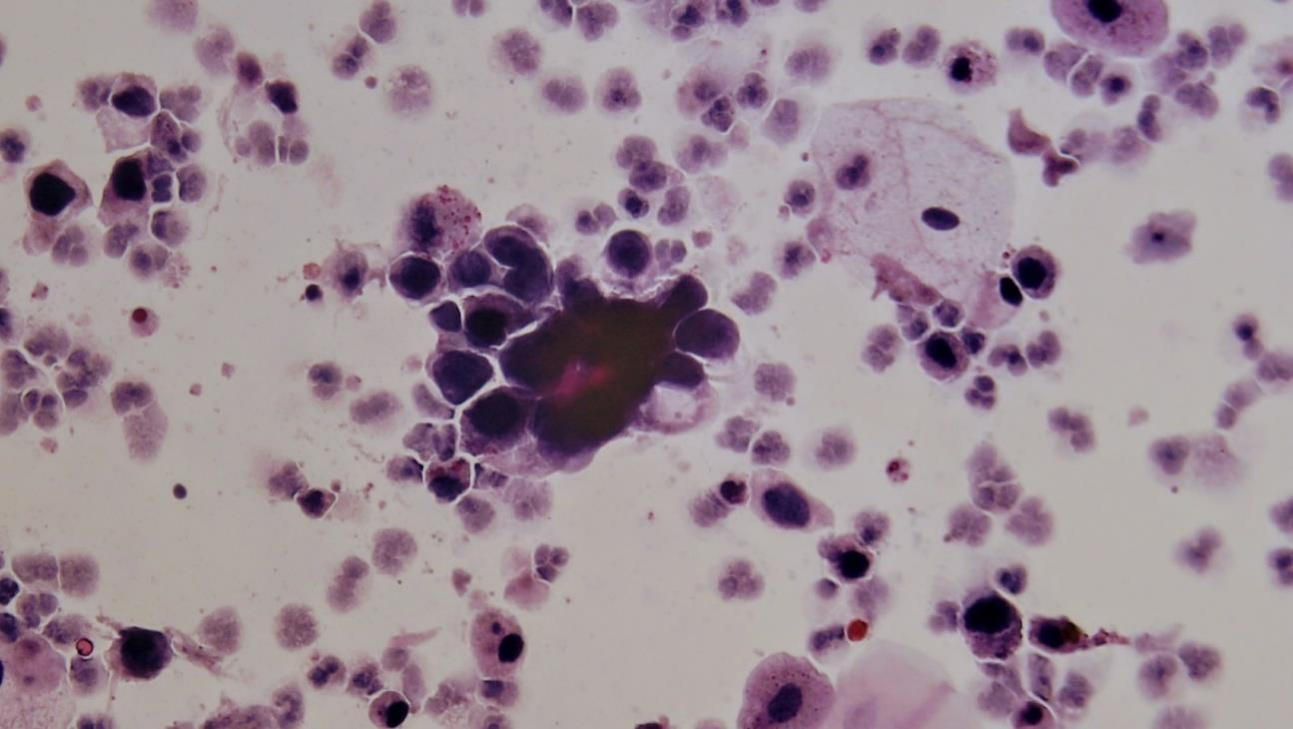
Liquid-based cytology. HGUC: High-grade urothelial carcinoma

**Fig. 2:**
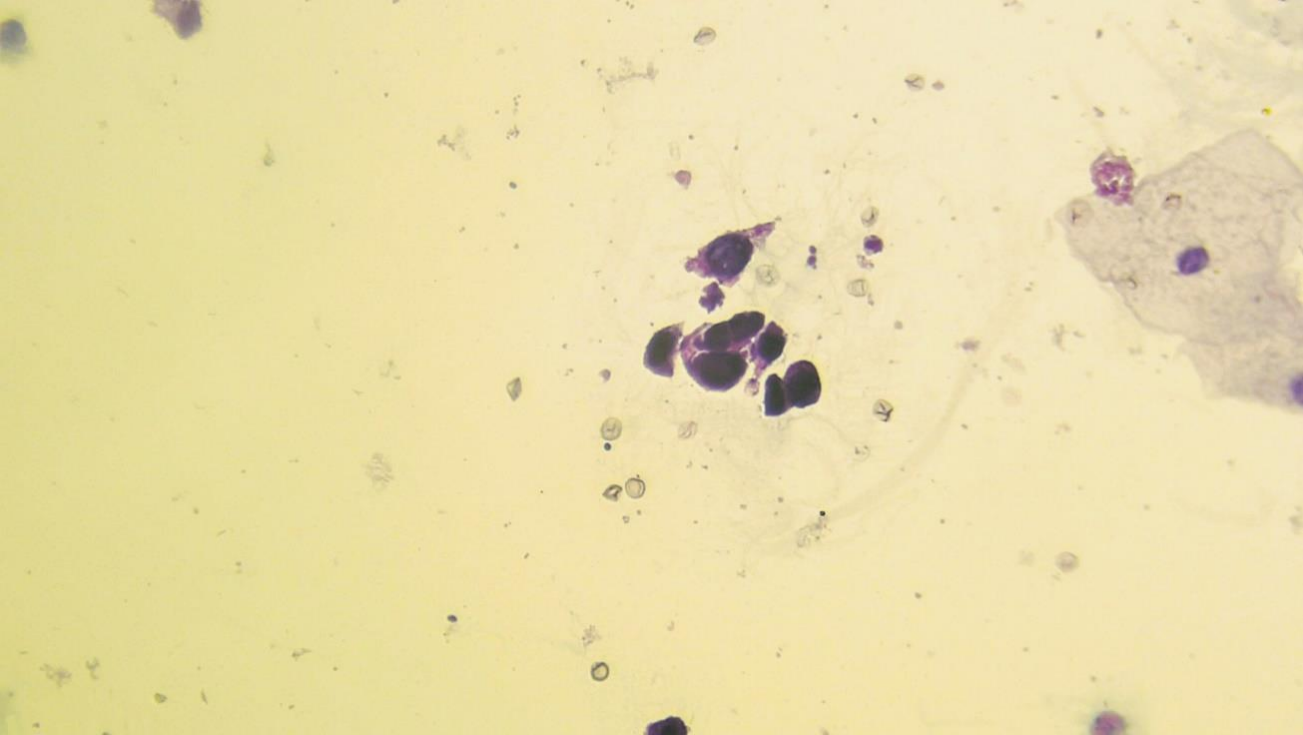
Liquid-based cytology. HGUC: High-grade urothelial carcinoma

HGUC: a cellular cytologic urine specimen with a minimum of five to ten viable malignant cells will qualify as HGUC (Figures 1 and 2).

**Fig. 3:**
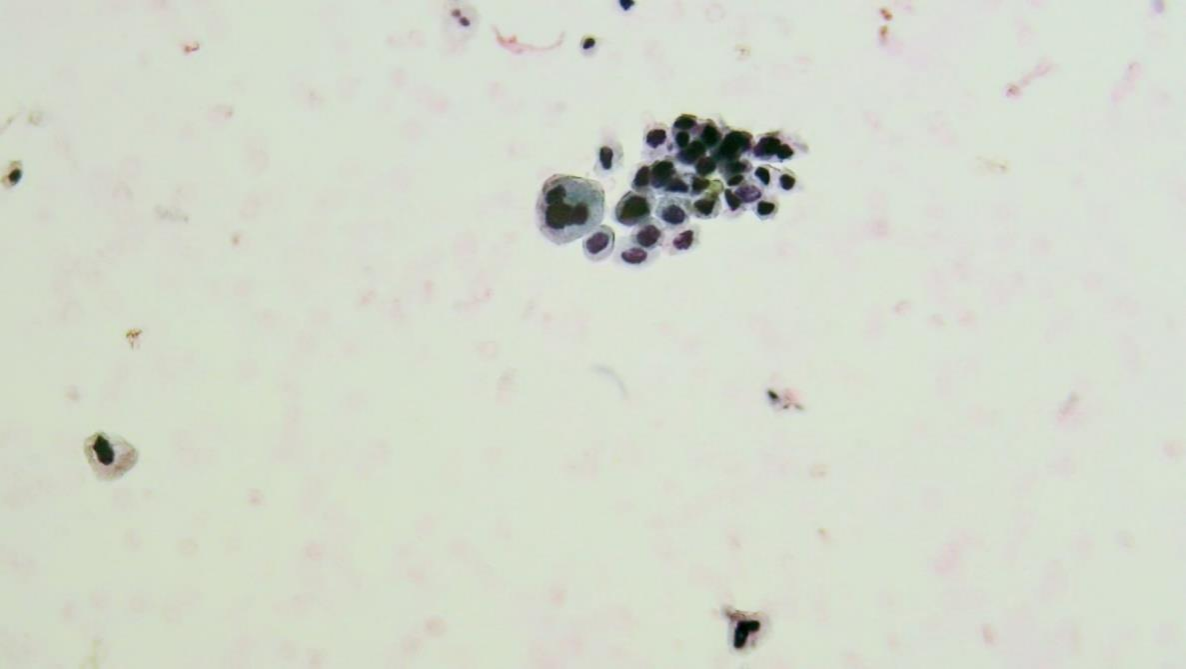
Liquid-based cytology. AUC: Atypical urothelial cells

**Fig. 4:**
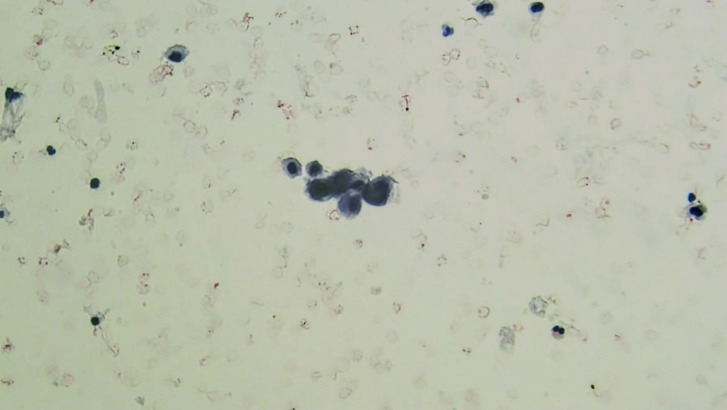
Liquid-based cytology. AUC: Atypical urothelial cells

Atypical Urothelial cells (AUC): cellular changes that fulfill the major criterion (nuclear-cytoplasmic ratio >0.5) and only one minor criterion (nuclear hyperchromasia, irregular nuclear membranes, irregular, coarse, and clumped chromatin) (Figures 3 and 4).

No urothelial atypia or malignancy (NUAM): if any of the following components are present: 

-Benign urothelial, glandular, and squamous cell

-Benign urothelial tissue fragments, sheets, or clusters

-Changes associated with lithiasis

-Viral cytopathic effect

-Post-therapy effect

Dysplasia: the urothelium shows markedly abnormal, enlarged, and hyperchromatic nuclei, though the degree of abnormality is somewhat less than in classic carcinoma in situ**.**

## Results

A total of 146 patients were included in the study. Patients’ mean age was 61 years; 102 of them were male, and 44 were female.

The findings of the LBC and DSC methods are presented in Table 1. Biopsy findings are presented in Table 2.

The findings of DSC methods were compared with biopsy results (Table 3). The comparison results of biopsy and liquid-based cytology are depicted in Table 4. The accuracy of cytology methods was evaluated according to biopsy reports. The sensitivity and specificity of these two methods were also calculated (Table 5).

We compared biopsy results with LBC and DSC results; findings are as follows:

- High-grade Transitional Cell Carcinoma (HGT-CC) in biopsy: 13 out of 146 cases were HGTCC, 10 of which were invasive transitional cell carcinoma (INTCC). Of these 13 HGTCC biopsy cases, five were reported as HGUC in LBC (two HGUC and three SHGUC cases in DSC); four were reported as SHGUC in LBC (two HGUC and two SHGUC cases in DSC, which had severe hematuria); two were reported as AUC Figures 1 and 2 in LBC (one NUAM and one AUC cases in DSC, which were accompanied by severe inflammation). Two were reported as NUAM both in LBC and DSC methods.
- Low-grade transitional cell carcinoma (LGTCC) in biopsy: 10 of the biopsy results were LGTCC. Of these 10 LGTCC biopsy results, five were AUC in LBC (three AUC and two NUAM cases, all accompanied by hemorrhage); three were LGUN in LBC, all of which were AUC in DSC; and two were NUAM in LBC and in DSC, one of which had severe hematuria.
- Carcinoma in situ in biopsy: two cases of carcinoma in situ, both reported as HGUC in LBC and DSC.
- Dysplasia in biopsy: three cases of dysplasia were reported in biopsies, which were AUC in LBC and NUAM in DSC.

Moreover, the comparison of LBC results with DSC and biopsy results is presented in Table 6.

In this study, the largest difference was reported in patients with a diagnosis of AUC in the LBC method. In these 11 cases, six cases were reported as negative in DSC, one case was unsatisfactory, and only four cases were reported as AUC in the DSC method.

The biopsies of these 11 patients revealed 10 positive and only one negative report (with reactive changes due to radiotherapy). Therefore, in the McNemar test, there was a significant difference between the LBC and DSC methods in AUC patients (*P*=0.016).

Kappa statistics revealed a significant agreement between LBC and DSC in negative biopsy cases (*P*<0.000), Kappa value=0.663. However, there was poor agreement between the two methods in positive biopsies (*P*>0.05).

**Table 1:** Frequency of liquid-base and direct smear cytology findings

Finding	LBC	DSC
NUAM	121 (82.9%)	**119 (81.5%)**
AUC	11 (7.5%)	**6 (4.1%)**
LGUN	3 (2.1%)	**2 (1.4%)**
SHGUC	4 (2.7%)	**5 (3.4%)**
HGUC	7 (4.8%)	**6 (4.1%)**
Total	100%	** 100%**

NUAM: No urothelial atypia or malignancy, AUC: Atypical urothelial cells, LGUN: Low-grade urothelial neoplasia, SHGUC: Suspicious for high-grade urothelial carcinoma, HGUC: High-grade urothelial carcinoma.

**Table 2:** Frequency of biopsy findings

Findings	Frequency	Percent
Negative	118	**80.8%**
Dysplasia	3	**2.1%**
CIS	2	**1.4%**
LGTCC	10	**6.8%**
HGTCC	13	**8.9%**
Total	146	**100**

CIS: Carcinoma in situ, LGTCC: Low-grade transitional cell carcinoma, HGTCC: High-grade transitional cell carcinoma.

**Table 3:** Biopsy and direct smear cytology comparing results

DSC finding	Frequency	Biopsy result: Negative (-) Positive (+)	
NUAM	119	109 (91.6%)	**10 (8.4%)**
AUC	6	2 (33%)	**4 (66%)**
LGUN	2	2 (100%)	**0**
SHGUC	5	0	**5 (100%)**
HGUC	6	0	**6 (100%)**
Unsatisfactory	8	7 (87.5%)	**1 (12.5%)**
Total	146	118 (80.8%)	**28 (19.2%)**

** -**Positive biopsy results consist of Dysplasia, CIS, LGTCC and HGTCC.

- NUAM: No urothelial atypia or malignancy, AUC: Atypical urothelial cells, LGUN: Low-grade urothelial neoplasia, SHGUC: Suspicious for high-grade urothelial carcinoma, HGUC: High-grade urothelial carcinoma.

**Table 4:** Biopsy and liquid-based cytology comparing results

LBC finding	Frequency	Biopsy result: Negative (-) Positive (+)	
NUAM	121	117 (96.7%)	**4 (3.3%)**
AUC	11	1 (9.1%)	**10 (90.9%)**
LGUN	3	0	**3 (100%)**
SHGUC	4	0	**4 (100%)**
HGUC	7	0	**7 (100%)**
Total	146	118 (80.8%)	**28 (19.2%)**

Positive biopsy results consist of Dysplasia, Carcinoma in situ, Low-grade transitional cell carcinoma, High-grade transitional cell carcinoma.

NUAM: No urothelial atypia or malignancy, AUC: Atypical urothelial cells, LGUN: Low-grade urothelial neoplasia, SHGUC: Suspicious for high-grade urothelial carcinoma, HGUC: High-grade urothelial carcinoma.

**Table 5:** Direct smear cytology and liquid-based cytology statistics

	DSC	LBC
Sensitivity	60.7%	**85.7%**
Specificity	98%	**99%**
Positive Predictive Value	89.4%	**96%**
Negative Predictive Value	91.3%	**96%**

**Table 6:** Comparing the result of liquid-based cytology with direct smear cytology and biopsy

LBC	DSC	Biopsy
	NUAM (N=6)	**Dysplasia (N=3)** **LGTCC (N=2)** **HGTCC (N=1)**
AUC (N=11)	AUC (N=4)	**LGTCC (N=2)** **HGTCC (N=1)** **Negative (N=1 , with reactive changes due to radiotherapy)**
	Unsatisfactory (N=1)	**LGTCC (N=1)**
HGUC (N=7)	HGUC (N=4)	**HGTCC (N=2)** **CIS (N=2)**
	SHGUC (N=3)	**HGTCC (N=3)**
LGUN (N=3)	LGUN (N=2) AUC (N=1)	**LGTCC (N=3)**
SHGUC (N=4)	HGUC (N=2) SHGUC (N=2)	**HGTCC (N=2)** **LGTCC (N=2)**

NUAM: No urothelial atypia or malignancy, AUC: Atypical urothelial cells, LGUN: Low-grade urothelial neoplasia, SHGUC: Suspicious for high-grade urothelial carcinoma, HGUC: High-grade urothelial carcinoma, CIS: Carcinoma in situ, LGTCC: Low-grade transitional cell carcinoma, HGTCC: High-grade transitional cell carcinoma.

## Discussion

Since 1939, screening of malignant neoplasms has relied on cytology. However, direct smear disadvantages (such as thick smear, overlapping cellular regions, low cellularity, inflammatory cells, red blood cells, and air bubble artifact) make the diagnosis difficult and result in low diagnostic sensitivity. Therefore, LBC has been introduced as a substitute for the conventional method and has been widely used over the past two decades (24-27). Several studies have compared DSC and LBC in fields other than gynecology. However, only a few studies have compared these two cytology techniques in urine specimens. In the analysis of 236 urine specimens conducted by Lee* et al.*, it was shown that the use of a ThinPrep-based liquid-based preparation method was useful to improve the quality of the slides and reduce the duration of the test, but the sensitivity, accuracy, and predictive value were not changed (27).

In another study, Koh* et al.* pointed out that the use of CellPrepPlus LBC for body liquids had a higher sensitivity and higher negative predictive value. The quality of the slides was better than DSC (28). Therefore, it is a useful diagnostic method in body fluid screening.

Conventional urine cytology methods include cell centrifugation (cytospin), Millipore filtration, and DSC. In the cytospin method, it may be associated with low cellularity and non-uniform cells throughout the slide or highly cellular smears with poor cell preparation. Contrary to urine cytology, in other fields (gynecology and non-gynecology), the conventional method of slide preparation generally has no problem with low cellularity. In contrast, highly cellular content requires more screening time due to the increased number of slides in each sample and analysis of different cell regions (28, 29).

In the LBC method, the cells are washed with a liquid and processed automatically. Compared to DSC, LBC shows less vague elements, while the cytological characteristics of cells are preserved. Therefore, LBC solves one of the major problems in urine cytology, which is the low cellularity (14).

In our study, a total of 146 samples were taken from patients suspected of bladder carcinoma and examined by both DSC and LBC. The sensitivity and specificity in DSC were 60.7% and 98% and in LBC were 85.7% and 99%, respectively. However, in Fakhrjoo* et al.*’s study, the sensitivity and specificity of DSC in the diagnosis of bladder tumors of 900 patients were 73% and 99%, respectively (20).

In 2009, Lu* et al.* compared CellPrepPlus LBC with the conventional smear in 713 patients. The diagnostic sensitivity for CellPrepPlus was 50% and higher than 37.5% for the conventional smear. The specificity of both preparations was 100% (17).

In our study, the positive predictive value was 89.4%, and the negative predictive value was 91.3% for DSC and 96% for LBC. We thought that the difference between other studies (20, 24) and ours is due to different processing methods, which preserve cytological characteristics of cells. We used ThinPrep for processing our specimens but, in most of the other studies, CellPrep was used. Other causes are various LBC diagnostic definitions, different sample sizes, and pathologists’ skills.

In our study, the biggest difference was in the AUC group (11 cases in LBC and six cases in DSC). In the LBC group, 10 out of 11 had positive biopsy results, and there was only one negative biopsy due to radiotherapy.

The McNemar test showed a significant difference between LBC and DSC (*P*=0.016). This difference was because of eliminating cellular debris, inflammatory cells, and red blood cells, as well as the preservation of cytological characteristics in the LBC method, which made the diagnosis easier. On the other hand, in AUC, the pathologist detected mild malignant cytologic changes; therefore, the omission of background elements was very helpful. However, in higher malignant grades, changes can be detectable in DSC, even in the presence of disturbing background elements.

Kappa statistics revealed a significant agreement between LBC and DSC in negative biopsy cases (*P*<0.000), Kappa value=0.663.

However, there was poor agreement between the two methods in positive biopsies (*P*>0.05). This finding shows that LBC is preferable to DSC in detecting positive cases and malignant cells due to lower background elements. LBC reduces unsatis-factory cases, which is not uncommon in the DSC method. In France, Piaton* et al. *(2005) examined 79 urine samples and found no significant difference between the LBC (ThinPrep) and DSC methods in the diagnosis of nuclear abnormalities (*P*=0.01) (30). In our study regarding nuclear abnormalities, there was no significant difference between the DSC and LBC methods, but the LBC method helped us to improve cell-free background; therefore, we managed to have lower AUC cases.

Kapoor* et al.* concluded that LBC offered better detection of malignant cells in the urine of patients with bladder tumors than DSC. Moreover, the detection of malignant cells by LBC was even better in the background of hematuria (31). These findings are similar to our results 

## Conclusion

This study shows higher sensitivity and higher specificity of Thinprep liquid-based cytology than direct smear cytology, especially in the diagnosis of Urothelial tumors with low-grade malignancy. LBC method can reduce AUC cases which is the waste-basket for pathologists. Furthermore, it lowers the unsatisfactory cases in DSC. In conclusion, although the LBC method costs more, its several advantages over the DSC method make it an appropriate alternate method to evaluate urinary samples.

## Conflict of Interest

The authors declared no conflict of interest.

## Funding

This study was supported by Mashhad University of Medical Sciences, Mashhad, Iran.
